# INVESTIGATING ADVERSE OUTCOMES OF LONG-TERM STEROID IMMUNOSUPPRESSION IN ELDERLY RENAL TRANSPLANT RECIPIENTS

**DOI:** 10.1093/geroni/igad104.2986

**Published:** 2023-12-21

**Authors:** John Johnson, Heather Stevenson, Muhammad Mujtaba, Michael Kueht

**Affiliations:** University of Texas Medical Branch at Galveston, Galveston, Texas, United States; University of Texas Medical Branch at Galveston, Galveston, Texas, United States; University of Texas Medical Branch at Galveston, Galveston, Texas, United States; University of Texas Medical Branch at Galveston, Galveston, Texas, United States

## Abstract

Steroids are widely used in renal transplantation to prevent allograft rejection; however, long-term steroid use is associated with adverse side-effects such as hyperglycemia and osteoporosis, which elderly patients are especially susceptible to. Our study analyzed the adverse effects of long-term steroid immunosuppression in elderly renal transplant recipients using the TriNetX database. TriNetX was used to perform a propensity-score matched case-control study of 5-year outcomes in elderly renal transplant recipients (≥ 65 yrs) who either underwent an early-steroid withdrawal (ESW) or steroid-continuous (SCI) maintenance immunosuppression regimen. ESW patients were maintained long-term (5 yr) on tacrolimus and mycophenolate and discontinued prednisone 7 days post-transplant. The SCI cohort was maintained on 5yr tacrolimus + mycophenolate + prednisone. Cohorts were matched on age, sex, race/ethnicity, BMI, diabetes, cardiovascular disease, and bone disorders. Primary outcomes included rejection, drug-induced diabetes, bone fractures, and malignancy 5 years post-transplant. There were 197 patients in each cohort after matching (ESW, SCI). SCI patients had significantly higher incidences of 5-year drug-induced diabetes (4.53% vs 15.73%, p < 0.01) when compared to ESW. There was no significant difference found for incidences of rejection (33.02% vs 33.91%, p = 0.86), bone fractures (12.27% vs 16.29%, p = 0.19), or malignancy (11.57% vs 15.78%, p = 0.26). Our study found that elderly SCI patients had significantly higher incidences of drug-induced diabetes when compared to ESW. More insight is needed into differences in average glucose, blood pressure, and cholesterol throughout the duration of the study and dosage differences within the SCI cohort.

